# Disease outbreak accompanies the dispersive structure of shrimp gut bacterial community with a simple core microbiota

**DOI:** 10.1186/s13568-018-0644-x

**Published:** 2018-07-18

**Authors:** Zhiyuan Yao, Kunjie Yang, Lei Huang, Xiaolin Huang, Linglin Qiuqian, Kai Wang, Demin Zhang

**Affiliations:** 1 School of Marine Sciences, Ningbo University, Ningbo, 315211 China; 2 Zhejiang Mariculture Research Institute, Wenzhou, 325005 China; 3 Collaborative Innovation Center for Zhejiang Marine High-efficiency and Healthy Aquaculture, Ningbo, 315211 China

**Keywords:** Gut bacterial community, Shrimp health state, Ecological processes, Core microbiota, Co-occurrence

## Abstract

Increasing evidence has emerged supporting a tight link between gut bacterial community and shrimp health. However, the knowledge about the variation of gut bacterial community, especially with different disease onset time, remains elusive. Here, healthy and diseased shrimps were collected at 3 disease-outbreak times (day 70, 80 and 85) to investigate the variation of gut bacterial community and its underlying ecological process with 16S rRNA gene amplicon sequencing. The gut bacterial community of diseased shrimp was distinct from the healthy one and temporally less stable, characterized by decreased alpha-diversity and dispersive structure. And its dominant ecological process experienced a transition with disease onset time, although deterministic process mainly governed the healthy gut bacterial assembly. In addition, the core microbiota of healthy shrimp gut harbored more diverse bacterial taxa with more cooperative interactions, while the diseased core microbiota showed opposite pattern with significantly higher abundance of opportunistic pathogens as well. These findings indicate that shrimp heath is highly relevant to the homeostasis of its gut bacterial community. Preservation and restoration of the bacterial community equilibrium could represent an effective strategy for shrimp disease prevention.

**Electronic supplementary material:**

The online version of this article (10.1186/s13568-018-0644-x) contains supplementary material, which is available to authorized users.

## Introduction

High frequency of disease outbreak is still one of the major bottle-necks of shrimp-culture industry development and often results in severe economic losses (Defoirdt et al. 2011; Thitamadee et al. 2016). The gut bacterial community has been considered as a crucial factor affecting animal health (Clemente et al. 2012; Li et al. 2017b; Xiong et al. 2015). Shrimp is directly and continuously in contact with the surrounding bacterioplankton and exposed to denser microbial invasions than terrestrial animals (De Schryver et al. 2014; Xiong et al. 2017). Its gut bacterial community is a comprehensive reflection of bacterioplankton community and could be more stable and reliable in indicating the host’s health state (Rungrassamee et al. 2014; Xiong et al. 2015). Shrimp has no adaptive immune system for natural protection which is different from mammals (Gonzalez-Santoyo et al. 2012), and thus the balance of shrimp gut bacterial community would play a more important role in disease resistance and health maintaining.

It has been proposed that gut bacterial community could guide the shrimp management in practice (De Schryver and Vadstein 2014; Zhu et al. 2016). Healthy and diseased shrimps have distinct gut bacterial communities and the deviations are closely correlated with disease severity (Cornejo-Granados et al. 2017; Xiong et al. 2015; Zhang et al. 2014). Xiong et al. (2017) demonstrated that shrimp disease initiation and severity can be accurately diagnosed using gut microbiota immaturity and bacterial indicators. Ecological processes are critical in shaping gut bacterial community (Burns et al. 2016). It has been revealed that the relative importance of deterministic processes decreases when shrimp disease occurs, which in turn make shrimp gut community more prone to invasion by alien strains (Xiong et al. 2017; Zhu et al. 2016). Therefore, understanding the association between shrimp gut bacterial community and health states and the underlying ecological processes is of essential interest from both commercial and scientific perspectives. Shrimp disease could occur at different time, while the gut bacterial community changes dramatically along with shrimp development (Huang et al. 2016; Rungrassamee et al. 2013; Xiong et al. 2017). So far, little is known about whether the variation of gut bacterial community related with disease outbreak would change with disease onset times. Hence, it is of great importance to untangle the variation of gut bacterial community between health states from that over shrimp development.

Another fundamental challenge in host-associated microbial ecology is to determine the extent to which microbial assemblages in a given host are shared among other hosts. Previous studies have shown that a subset of gut bacterial community is continuously present and can be found in other hosts, a concept often referred to as “core microbiota” (Roeselers et al. 2011; Wong et al. 2013; Zhang et al. 2015). The existence of core microbiota has been demonstrated in many hosts (e.g. mammals, insect and fish) (Sekelja et al. 2011; Shetty et al. 2017; Turnbaugh et al. 2009), which would maintain the homeostasis and functional stability of bacterial community necessary for a healthy gut. Until now, the knowledge about the core microbiota of shrimp gut is scarce although identifying core microbiota of shrimp gut is essential in defining a “healthy” gut bacterial community and contributes to revealing keystone species in the community (Roeselers et al. 2011). The relatively consistent environmental and dietary parameters inherent in shrimp aquaculture facilities contribute to exploring the core microbiota in shrimp gut (Wong et al. 2013), providing opportunities to determine the extent such cores are affected by health state.

In this study, healthy and diseased shrimps were collected at 3 disease-outbreak times within same culture duration. We applied 16S rRNA gene amplicon sequencing to investigate (i) the variation of gut bacterial community related with health state at different disease onset time; (ii) the underlying ecological assembly processes of gut bacterial community correlated with shrimp health state; (iii) the presence of core microbiota in shrimp gut and its relationship with health state.

## Materials and methods

### Experimental design and sample collection

The shrimp ponds investigated in this study are located in Ningbo, China (29°32′N, 121°31′E). Shrimp (*Litopenaeus vannamei*) juveniles were introduced into the ponds on 27 March, 2014. The management of the test ponds is consistent with previous study (Xiong et al. 2015) and the shrimp stocking density is 360,000 inds/pond. Disease occurred in different ponds at 70, 80 and 85 days after inoculation, which caused massive mortality of shrimp. All the diseased shrimps had usual pathological symptoms of the disease, such as lethargy, empty gut and pale white aqueous hepatopancreas and stopped eating, which are same as the symptoms of acute hepatopancreatic necrosis disease (AHPND). According to shrimp health state, the shrimp ponds were categorized into healthy (HI) and diseased (DI) group. Five shrimps were collected from each pond and combined to form a biological sample representing a given pond. There was no overlap of the diseased shrimp ponds across 3 disease onset times. Fifty-eight shrimp samples (31 healthy versus 27 diseased) were collected from 7 June to 21 June (corresponding to 70, 80 and 85 days after inoculation). The detailed sampling information is showed in Additional file 1: Table S1. The collected shrimp samples were transferred to the laboratory in an ice-box within 2 h.

### DNA extraction, bacterial 16S rRNA gene amplification and Illumina MiSeq sequencing

The treatment of shrimp samples and sequencing were same as previous report (Xiong et al. 2015). Total DNA was extracted using QIAamp DNA Mini Kit (Qiagen, GmbH, Hilden, Germany) according to the manufacturer’s protocols. The extracted DNA was quantified with NanoDrop ND-1000 spectrophotometer (NanoDrop Technologies, Wilmington, USA) and stored at − 80 °C prior to amplification. PCR primers 338F (5′-ACTCCTACGGGAGGCAGCA-3′) and R806 (5′-GGACTACHVGGGTWTCTAAT-3′) with dual barcode sequences were used to amplify the V3–V4 region of 16S rRNA gene. Each DNA sample was amplified in triplicate (in a 20 μL reaction system) and pooled to minimize reaction-level PCR bias under the following conditions: initial denaturation at 95 °C for 3 min, followed by 28 cycles of denaturation at 95 °C for 30 s, annealing at 55 °C for 30 s and extension at 72 °C for 45 s, with a final extension at 72 °C for 10 min. PCR products of each sample were combined and purified with PCR fragment purification kit (TaKaRa Biotech, Japan). Equimolar amount of PCR products from each sample were combined in a single tube and ran on a Miseq sequencer (Illumina, San Diego, USA).

### Processing of sequencing data

Raw data were analyzed by combined QIIME v.1.9.1 (Caporaso et al. 2010) and USEARCH v.6.1 (Edgar et al. 2011) pipelines. Paired-end reads were merged with fast length adjustment of short reads (FLASH) using the default setting (Magoč and Salzberg 2011). Subsequently, the reads were truncated at the site of more than three bases that received Phred scores (Q) < 20, and the reads with lengths less than 75% of the total read length were discarded (Bokulich et al. 2013). OTU clustering was performed with pick_open_reference_otus.py script using the SUMACLUST (Mercier et al. 2013) and SortMeRNA methods (97% cutoff) (Kopylova et al. 2012). The most abundant sequence of each OTU was selected as the representative sequence and then was taxonomically assigned against SILVA_128 database (https://www.arb-silva.de/documentation/release-128/). There are 2,015,676 reads matching against 16S SILVA database, while 9567 reads were unclassified due to chimera. A phylogenic tree was generated from the filtered alignment using FastTree (Price et al. 2009). All Archaea and Chloroplast sequences were removed, as were the other sequences that could not be assigned to bacteria. Singletons were also discarded. Rarefaction curves were constructed for each individual sample showing the number of observed OTUs (Additional file 1: Figure S1). To correct for varying sampling efforts, data were randomly rarefied at the same sequence depth (26,100 sequences) corresponding to the smallest sequencing effort for any of the samples for downstream analyses. Alpha-diversity and beta-diversity estimates were calculated by rarefaction at 26,100 reads per sample using QIIME, respectively, with respect to multiple indices (number of observed species, Shannon–Wiener index, and phylogenetic diversity), Bray–Curtis, weighted and unweighted Unifrac distance between samples. The sequence data were deposited in the NCBI (https://www.ncbi.nlm.nih.gov/) and are available under accession number SRP131736.

### Statistical analysis

One-way analysis of variance (ANOVA) was applied to investigate the impact of disease outbreak on alpha-diversity indices and the relative abundance of phylum/class abundances in each sampling day using SPSS 16.0. Principal coordinate analysis (PCoA) based on Bray–Curtis, weighted and unweighted Unifrac distance was applied to evaluate the overall differences in bacterial community structure. A permutational multivariate analysis of variance (PERMANOVA) was applied to evaluate the differences in gut bacterial communities corresponding to health state, time and their interaction based on Bray–Curtis and weighted and unweighted Unifrac distance metrics using the ‘ADONIS’ function of R (Anderson 2001). To find representative phylotypes associated with shrimp health state, a similarity percentage (SIMPER) analysis was applied to screen OTUs that contribute more than 1% dissimilarity each based on Bray–Curtis dissimilarity in the bacterial communities of different health state at each time point using PAST (Clarke 1993). Net relatedness index (NRI) values were computed based on phylogenetic tree, while the pairwise phylogenetic turnover between communities (βMNTD) was calculated as the mean nearest taxon distance metric (Kembel 2010). Furthermore, to infer community assembly processes, we calculated the β-nearest taxon index (βNTI), which is the difference between observed βMNTD and mean of the null distribution of βMNTD normalized by its standard deviation. Briefly, if βNTI values are βNTI > 2 or βNTI < − 2, deterministic processes are important in shaping the community composition across all sites, whereas if βNTI values are between − 2 and 2, stochastic processes will play an important role. All NRI and MNTD analyses were conducted using ‘Picante’ package in R (Kembel et al. 2010).

The phylogenetic core microbiota was screened out using PhyloCore, which uses a phylogeny-based algorithm to identify core taxa at OTU level (Ren and Wu 2016). For each internal node, PhyloCore calculated a prevalence value, defined as the cumulative presence of all its descendant OTUs. The prevalence threshold was 1.0 in this study. A node is considered a core node if its prevalence is above the threshold. Besides, OTUs with relative abundances lower than the threshold (1%) in a sample will be considered absent. To further test the existence of core microbiota occurred in healthy and diseased shrimp gut, we identified the OTUs present in specific health state (operationally defined here as a ‘healthy core’ or ‘diseased core’ according to its corresponding health state). The core OTUs occurred both in healthy and diseased core microbiota were defined as the ‘shared core’. Furthermore, the co-occurrence/interaction patterns among the detected core OTUs were explored in network analysis using CoNet 1.1.1 (Faust and Raes 2012, 2016). Pair-wise associations among OTUs were calculated using the Pearson, Spearman, Kendall, Bray–Curtis and Kullback–Leibler correlation methods simultaneously. The *p*-values were then merged using Brown’s method (Brown 1975) and corrected for multiple testing with the Benjamini–Hochberg (1995) procedure. Edges supported by at least three correlation methods with adjusted *p*-values below 0.05 were retained. A final network was then visualized in Cytoscape (version 3.5.1; Shannon et al. 2003; Smoot et al. 2011). Network Analyzer tool was used to calculate network topology parameters.

## Results

### Alpha-diversity, composition and structure of shrimp gut bacterial community

**Fig. 1:**
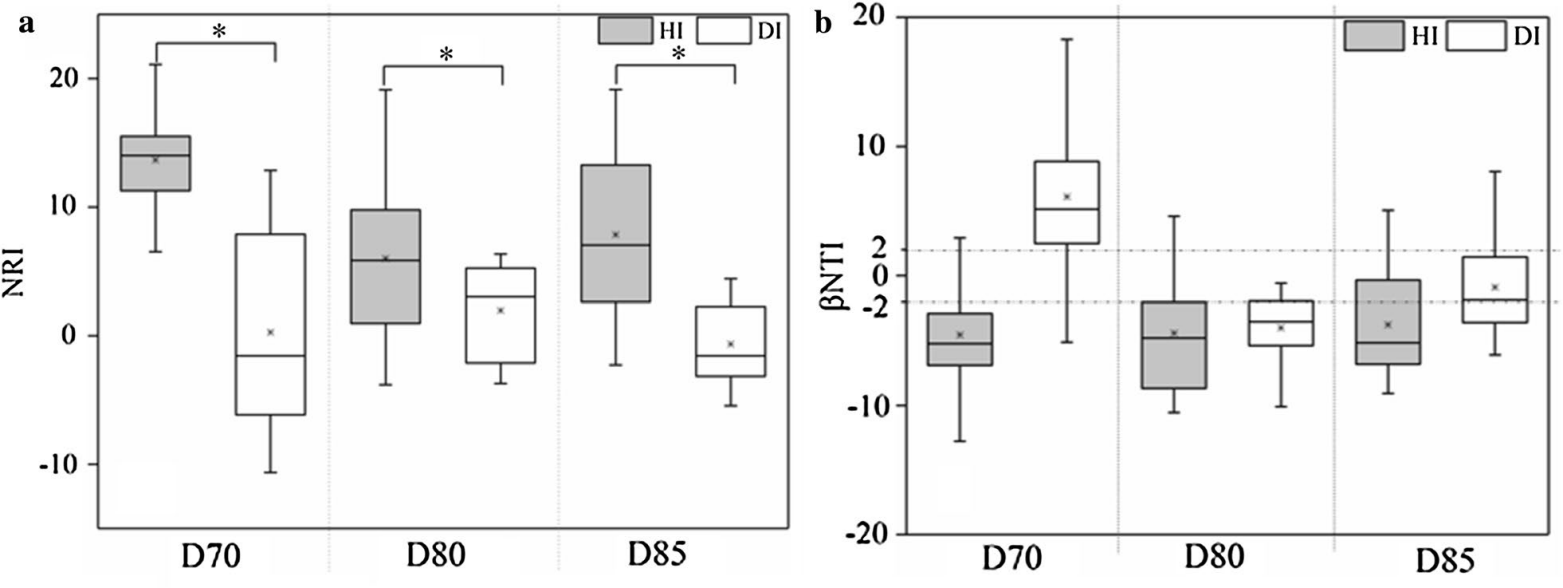
The net relatedness index (NRI) (**a**) and β-nearest taxon index (βNTI) (**b**) of the shrimp gut samples under different health state across three sampling days. Horizontal dashed lines in **b** indicate upper and lower significance thresholds at βNTI = + 2 and − 2, respectively. *HI* healthy gut, *DI* diseased gut. Significant differences were indicated by the asterisk (*P < 0.05) based on one-way analysis of variance. Lines at the top, bottom, and middle of the box correspond to the 75th, 25th, and 50th percentiles (median), respectively. The asterisk in the box represents the mean value

**Fig. 2:**
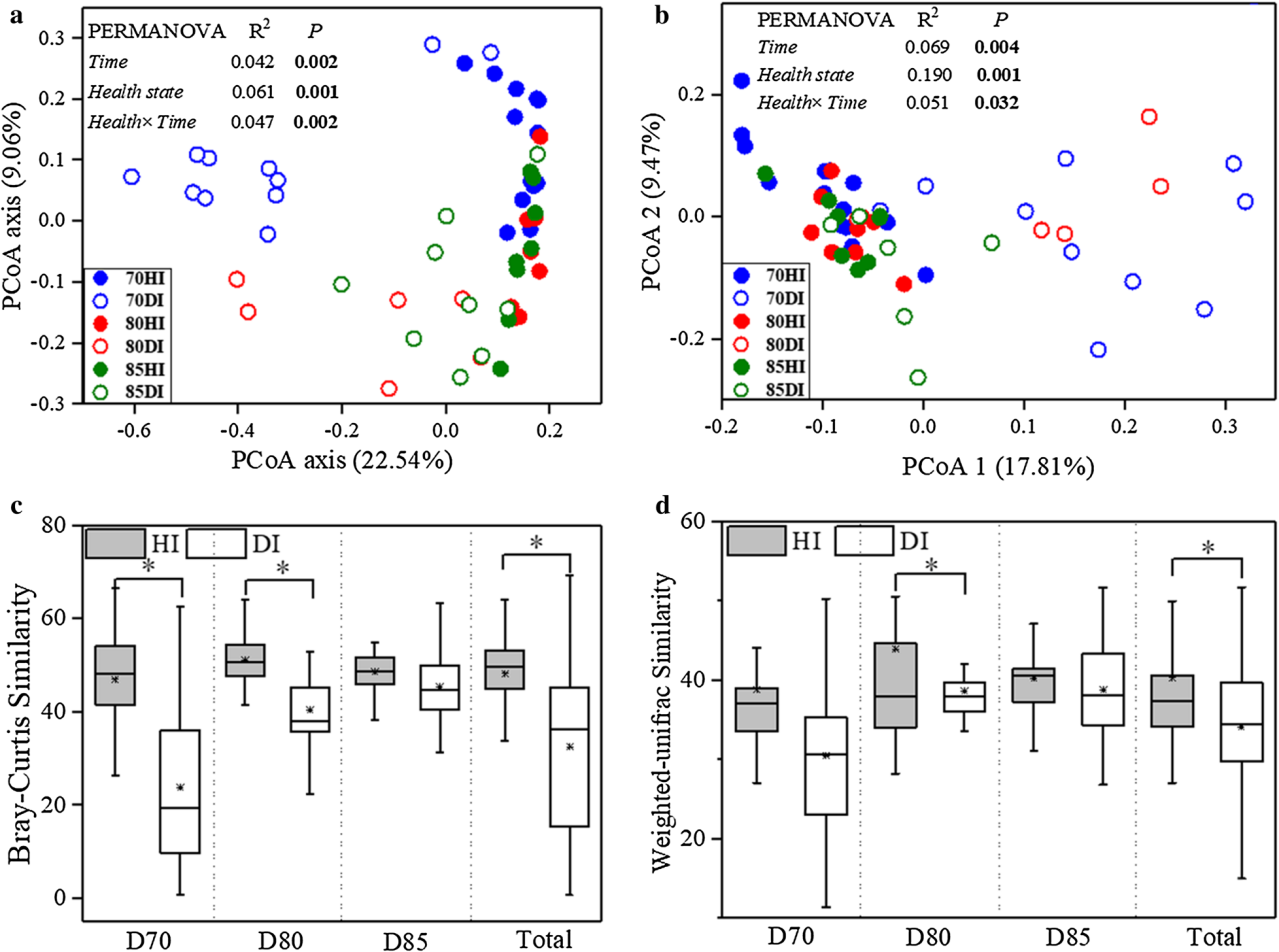
Principal coordinate analysis (PCoA) plots of community dissimilarities based on Bray–Curtis (**a**) and weighed Unifrac distance (**b**) between healthy and diseased shrimp gut across three sampling days. Sampling days exhibited with distinct colors (Blue: Day 70; Red: Day 80; Green: Day 85) and health state showed with the solid (Healthy) and hollow (Diseased). Permutational Multivariate Analysis of Variance (PERMANOVA) was used to test the significance of time, health state and their interaction in community variation at each day. Box plots of healthy and diseased gut bacterial communities based on Bray–Curtis (**c**) and weighed Unifrac (**d**) similarity within each and all sampling day. *HI* healthy gut; *DI* diseased gut. Significant differences were indicated by the asterisk (*P < 0.05) based on one-way analysis of variance. Lines at the top, bottom, and middle of the box correspond to the 75th, 25th, and 50th percentiles (median), respectively. The asterisk in the box represents the mean value

The bacterial α**-**diversity indices were considerably lower in diseased shrimp (DI) than in the healthy control (HI) (Additional file 1: Figure S2). Furthermore, the phylotypes in healthy shrimp gut were more closely related to each other than would be expected as assessed by NRI index, while the phylotypes in diseased shrimp gut was less related (Fig. 1a). The relative abundances of some dominant taxonomic groups changed apparently with disease outbreak, the relative abundances of *Alphaproteobacteria* and *Actinobacteria* in HI group were greater than those of DI group, while the relative abundance of *Gammaproteobacteria* was significantly lower than that of DI group (Additional file 1: Figure S3). A PCoA ordination biplot revealed that shrimp gut samples were clustered by health state at both taxonomic and phylogenetic scale (Fig. 2a, b; Additional file 1: Figure S4). Accordingly, PERMANOVA confirmed that health state showed stronger explanatory power to the variation in gut bacterial community than time did (Bray–Curtis: health state, R^2^ = 0.061, time, R^2^ = 0.042; weighted Unifrac: health state, R^2^ = 0.190, time, R^2^ = 0.069: unweighed Unifrac: health state, R^2^ = 0.085, time, R^2^ = 0.100) (Fig. 2a, b; Additional file 1: Figure S4). The diseased group dispersively distributed on the PCoA Plot while the healthy group concentrated in one side of the plot (Fig. 2a, b; Additional file 1: Figure S4). Consistently, the average similarities among healthy gut bacterial communities were significantly higher than the diseased except at day 85, which indicated a closer association between healthy gut bacterial communities (Fig. 2c, d). Moreover, the diseased gut bacterial community tended to be more different along with time than the healthy ones (Fig. 2c, d).

### Discriminatory assemblages for distinct health state

**Fig. 3:**
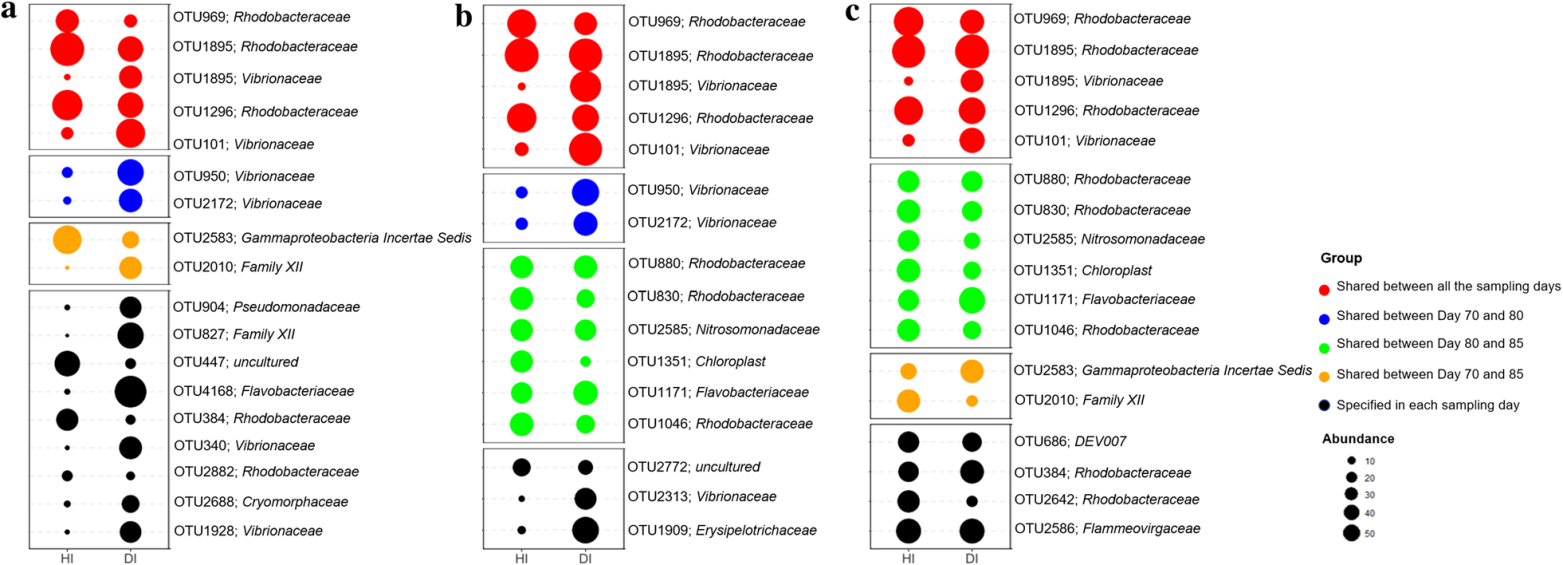
OTUs (relative abundance > 1%, classified at family level) that exhibited significant difference in relative abundance of healthy (HI) and diseased gut (DI) at day 70 (**a**), 80 (**b**) and 85 (**c**). The abundances of the indicator taxa were quarter square root transformed and showed with the size of bubbles from high to low

Based on SIMPER analysis, 18, 16 and 17 OTUs with significant (*P* < 0.05) changes in their relative abundances between different health states were identified as discriminatory assemblages for the health state at day 70, 80 and 85, respectively (Fig. 3). Totally, there were discriminatory 41 OTUs across all three sampling days with 5 (3 affiliated to *Rhodobacteraceae* and 2 to *Virbrionaceae*) of them were shared for all three sampling days. The majority (10) of OTUs which were enriched in healthy shrimp were affiliated to *Rhodobacteraceae.* In contrast, a number (7) of OTUs affiliated to *Virbrionaceae* were overrepresented in the diseased shrimps compared with the healthy ones (Fig. 3).

### Ecological processes governing the assembly of shrimp gut bacterial community

βNTI values were calculated to evaluate how disease emergency influences the ecological processes in the assembly of gut bacterial community. The ecological processes that regulate the assembly of gut bacterial community were consistent over time for healthy shrimp (Fig. 1b). A significant deviation (βNTI < − 2) indicated the dominance of homogeneous selection (e.g. host filtering) in healthy shrimp (Fig. 1b) across all three sampling days. By contrast, the ecological process of diseased shrimp varied with disease-outbreak time. It experienced a transition from heterogeneous selection (βNTI > + 2 at day 70) to homogeneous selection (βNTI < − 2 at day 80) and then to stochastic process (− 2 < βNTI < + 2 at day 85) across three disease onset times (Fig. 1b).

### Phylogenetic core microbiota

**Fig. 4:**
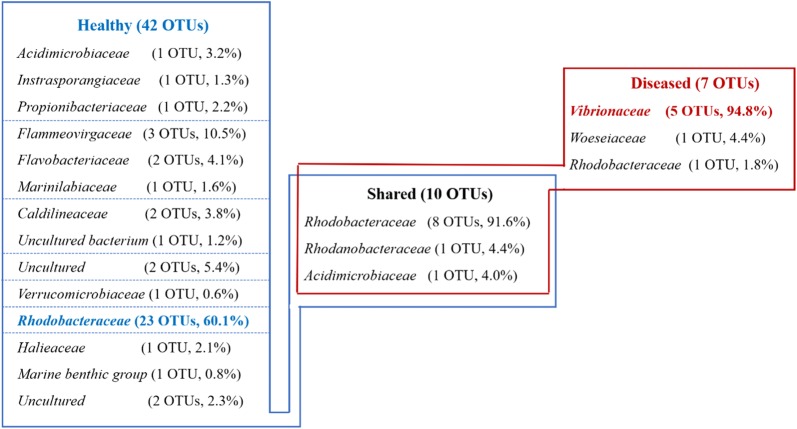
Taxonomy of core OTUs assigned to health state clusters at the family level and their abundances and average relative abundances in core microbiota of each category

The core microbiota was also markedly different from each other. The unique healthy core was mainly dominated by *Rhodobacteraceae* (23 OTUs, relative abundance 60.1%) and *Flammeovirgaceae* (3 OTUs, relative abundance 10.5%), while the unique diseased core was dominated by *Vibrionaceae* (5 OTUs, relative abundance 94.8%) (Fig. 4). However, the diseased core was more simple and stable than the healthy one across 3 sampling days, which was implied by ADONIS analysis (HI group: R^2^ = 0.11, *P* = 0.024; DI group: R^2^ = 0.04, *P* = 0.922). Ten OTUs were shared between two distinct core microbiota (Fig. 4). The shared core contained more than half of the OTUs that appeared in the unique diseased core, while less than 20% of the unique healthy core (Fig. 4). Furthermore, the shared core was composed primarily of OTUs affiliated to *Rhodobacteraceae* (8 OTUs, relative abundance 91.6%) (Fig. 4).

### Co-occurrence patterns of the phylogenetic core microbiota

**Fig. 5:**
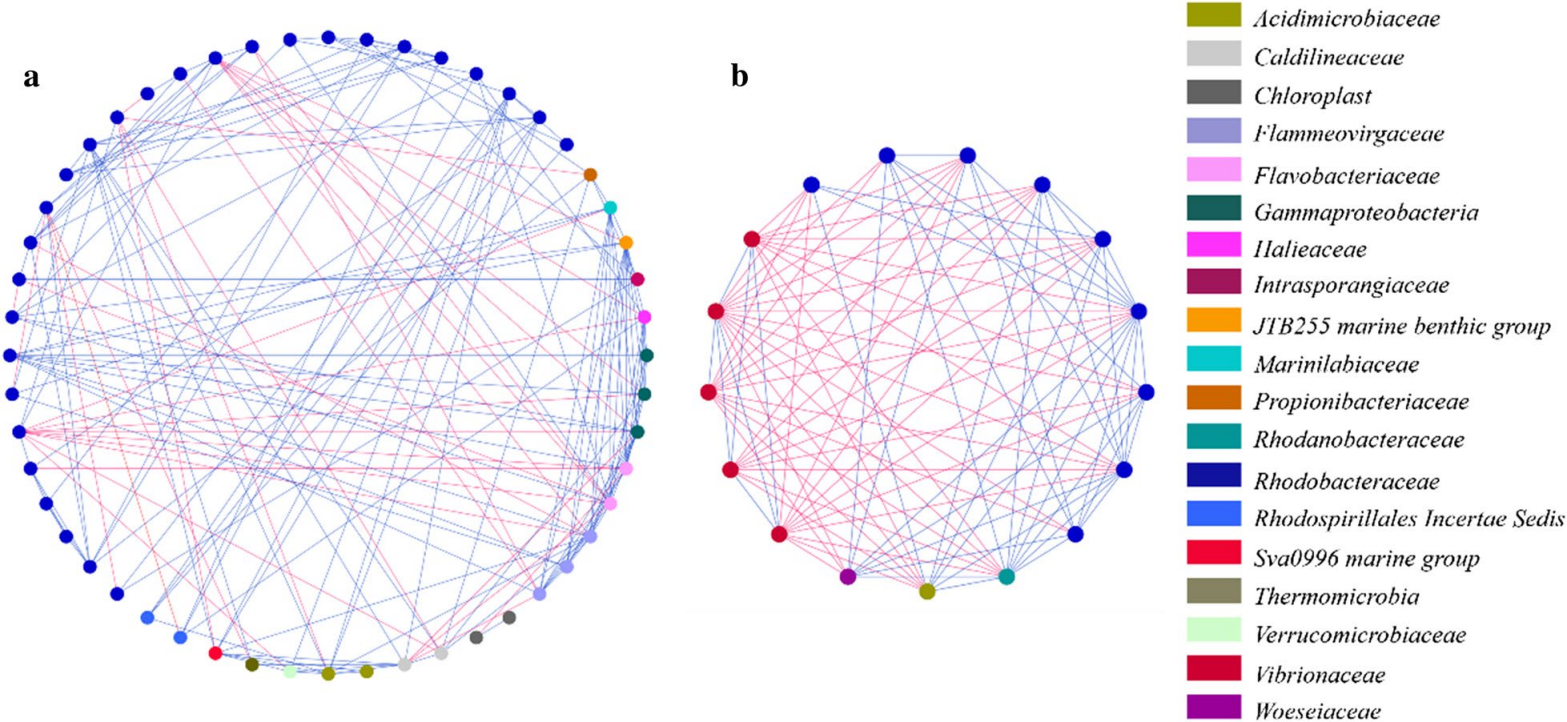
Network analysis revealing co-occurrence patterns of healthy (**a**) and diseased (**b**) core microbiota. Each node represents a bacterial OTU. The colors of the nodes indicate the OTUs affiliated to distinct major families. A blue edge indicates a positive correlation, whereas a red edge indicates a negative correlation between nodes

To evaluate the effect of disease outbreak on species-to-species interaction within the core microbiota, bacterial network analyses were conducted (Table 1, Fig. 5). The co-occurrence network of healthy core consisted of 52 nodes and 179 edges, which were apparently more than those of the diseased one (17 nodes and 97 edges) (Table 1). And the network density, centralization of betweenness and average clustering coefficient were lower than those of the diseased core, while characteristic path length exhibited the opposite trend (healthy core: 2.496; diseased core: 1.287) (Table 1). In addition, the percentage of exclusion interaction was much higher in diseased core than that in the healthy one (53.0% versus 19.0%, respectively; Table 1). Interestingly, all the exclusion interactions involved with the *Vibrionaceae* mainly connected with *Rhodobacteraceae* in the diseased core, while there was no exclusion interaction within members of *Vibrionaceae* OTUs (Fig. 5).

**Table 1:** Overall characteristics of the microbial networks of core microbiota corresponding to health state

	Healthy	Diseased
Node	52	17
Edge	179	97
Network density	0.135	0.713
Clustering coefficient	0.387	0.802
Network centralization	0.165	0.325
Characteristic path lengths	2.496	1.287
Co-presence links	145	47
Exclusion links	34	50
Exclusion links/total links	0.19	0.53

## Discussion

### Disease outbreak was accompanied with decreased α-diversity and dispersive taxonomic structure of gut bacterial community

Diversity is important in all ecosystems for promoting stability and performance. Gut microbial diversity may be considered as a biomarker of host health and metabolic capacity (Li et al. 2017a, b). This study provided further evidence that the alpha diversity of gut bacterial community significantly decreased in the diseased shrimp regardless of the outbreak time (Additional file 1: Figure S2), which is consistent with the previous studies suggesting that the disease outbreak likely accompany the decreased gut bacterial diversity (Cornejo-Granados et al. 2017; Xiong et al. 2015, 2017). Thus, the diversity of gut bacterial community is an important parameter in host–microbe symbiosis and may be associated with diseases. The gut bacterial communities of diseased shrimp varied dispersively and temporally faster and shared lower similarity with each other (Fig. 2 and Additional file 1: Figure S4), which reinforced the result of α-diversity that the diseased gut bacterial community was less stable than the healthy one. A similar phenotype of shrimp disease may be related with different microbial composition, which has been demonstrated in mammals that patients with recurrent *Clostridium difficile*-associated diarrhea (CDAD) harbor greatly distinct gut microbiota from each other (Chang et al. 2008). The instability of diseased gut bacterial community might also make it more sensitive to invading pathogens, as evidenced by the higher occurrence frequency of opportunistic pathogens (e.g. *Vibrio* and *Pseudomonas*) in diseased shrimp gut (Figs. 3 and 4). The characteristics of gut bacterial community of the diseased shrimp indicates that shrimp disease emergence might be highly associated with the dysbiosis of gut bacterial community. This means that apart from specific microbial taxa, shrimp disease might also be attributed to a complex community variation, which is consistent with the result of Xiong et al. (2017).

### The ecological assembly processes governing the bacterial community in diseased shrimp gut changed with disease outbreak time

Disease emergency could result in marked changes in the shrimp gut bacterial community, which could be reflected in the changes in the phylogenetic structure of the community (Pérez-Valera et al. 2017). The healthy gut held a more clustered phylogenetic community than the diseased one (Fig. 1a), which suggests that the health state exerts a stronger filtering on the gut assemblages than other factors (Stephens et al. 2016; Zhou and Ning 2017). And the concurrent changes in richness and phylogenetic diversity revealed that the missing species resulting from disease occurrence were phylogenetically clustered (Fig. 1a and Additional file 1: Figure S2). Furthermore, healthy shrimp exerts homogeneous selection on exogenous colonizers (Fig. 1b), thus contributing to similar community compositions (Fig. 1a) (Martınez et al. 2015). This was supported by the view that deterministic processes drive the assembly of the gut bacterial community of healthy aquatic animals (e.g. fish and shrimp) (Burns et al. 2016; Xiong et al. 2017; Yan et al. 2016). On the contrary, the ecological process governing the diseased shrimp transit with the disease onset time, from homogeneous filtering to heterogeneous, and then to stochastic process (Fig. 1b). The disease severity of diseased shrimp in this study might differ from each other, which might be another reason of the dispersive structure of diseased shrimp guts (Fig. 2 and Additional file 1: Figure S4) and the transition of ecological process (Fig. 1b). A more detailed characterizing of disease is needed to deeply reveal the understanding mechanisms for the variation in shrimp gut bacterial community with disease outbreak.

### Shrimp gut core microbiota changed greatly with disease outbreak

Despite the highly individual-specific profiles of gut bacterial communities, we identified few species (OTUs) contributing towards a core community corresponding to health state. Although the structure of gut bacterial communities diversified and became unstable along with disease outbreak (Fig. 2 and Additional file 1: Figure S4), the core microbiota of diseased shrimp gut became less diverse and more stable. The smaller core in diseased shrimp suggests loss of health-specific core taxa and potentially more heterogeneous bacterial community as a proxy of dysbiosis (Salonen et al. 2012). Moreover, a higher average clustering coefficient and a lower centralization of betweenness in diseased core indicated more redundancy in bacterial networks, implying that the loss of one or several keystone species may not affect the stability of the diseased core microbiota (Table 1) (Ruiz-Moreno et al. 2006).

The major compositions of the healthy core microbiota were OTUs affiliated to *Rhodobacteraceae*, while OTUs affiliated to *Vibrionaceae* were unique and dominant in diseased core (Fig. 4). Besides, OTUs affiliated to *Rhodobacteraceae* and *Vibrionaceae* were also specific in healthy and diseased shrimp gut bacterial community, respectively (Fig. 3), which is consistent with previous results (Chen et al. 2017; Zhu et al. 2016). Positively and negatively correlated co-occurrence patterns indicated by co-presence and exclusion links, respectively, could be interpreted in terms of either niche preferences or ecological interactions (Barberan et al. 2012; Faust and Rase 2012; Pascual-Garcıa et al. 2014). The higher ratio of exclusion link in diseased core indicated that species have more various niche and/or involved more in interactions such as amensalism, competition or predation, but more cooperative activities occurred in healthy shrimp (Faust and Raes 2012). And it is interesting that all the exclusion links in diseased core occurred between OTUs of *Rhodobacteraceae* and *Vibrionaceae* (Fig. 5), demonstrating the antagonistic relationship between them (Balcázar et al. 2006; Cude et al. 2012; Slightom et al. 2009). It has been reported that *Rhodobacteraceae* persists across shrimp growth stages, and likely form the gut core microbiota (Huang et al. 2016). Moreover, most of them have a de novo pathway for vitamin B12 synthesis, which has been shown to be essentials for shrimp diet (Li et al. 2017a; Lim and Akiyama 1995). This means that *Rhodobacteraceae* may play a health-promoting role on shrimp gut and the balance between *Rhodobacteraceae* and opportunistic pathogens (*Vibrionaceae*) might be critical in maintaining homeostasis of gut bacterial community and keeping shrimp health. Hence, *Rhodobacteraceae* may be a great source as probiotics in shrimp culture, within which some strains (*Roseobacter* clade) have been practically applied in aquaculture (Balcázar et al. 2006; Ruiz-Ponte et al. 1999).

Taken together, concomitant with disease outbreak the gut bacterial community experienced a series of variation: α-diversity decreased, and taxonomic structure of gut bacterial community became dispersive and temporally less stable while the core microbiota became more simple and stable. The ecological process in healthy shrimp gut was consistently dominated by homogeneous deterministic factors while it switched among homogeneous, heterogeneous and stochastic factors in diseased shrimp gut. These findings indicate that shrimp heath is highly relevant to the homeostasis of its gut bacterial community. Preservation and restoration of the bacterial community equilibrium could represent an effective strategy for shrimp disease prevention.

## Additional file

**Additional file 1: Table S1.** The average body length and the weight of the shrimp under different health state across three sampling days. **Figure S1.** Rarefaction curves of individual shrimp samples. Rarefaction curves were assembled showing the number of OTUs, defined at the 97% sequence similarity cut-off, relative to the number of total sequences. The dashed vertical line indicates the number of sequences subsampled from each sample to calculate alpha diversity estimates. **Figure S2.** The alpha-diversity indices (Shannon index and number of observed species, per 26100 sequences of the shrimp gut samples under different health state across three sampling days. HI: healthy gut; DI: diseased gut. Significant differences were indicated by the asterisk (*, *P* < 0.05) based on one-way analysis of variance. Lines at the top, bottom, and middle of the box correspond to the 75th, 25th, and 50th percentiles (median), respectively. The asterisk in the box represents the mean value. **Figure S3.** Relative abundance of the dominant bacterial phyla (>3%) or classes (*Proteobacteria*) under different health state across three sampling days. Each bar represents the mean ± standard deviation. Significant differences were indicated by the asterisk (*, *P* < 0.05; **, *P* < 0.01) based on one-way analysis of variance. **Figure S4.** Principal coordinate analysis (PCoA) plots of community dissimilarities based on unweighed Unifrac distance between healthy and diseased shrimp gut across three sampling days. Sampling days exhibited with distinct colors (Blue: Day 70; Red: Day 80; Green: Day 85) and health state showed with the solid (Healthy) and hollow (Diseased). Permutational Multivariate Analysis of Variance (PERMANOVA) was used to test the significance of time, health state and their interaction in community variation at each day. HI: healthy gut; DI: diseased gut.

## Abbreviations

AHPND: acute hepatopancreatic necrosis disease
ANOVA: one-way analysis of variance
DI: diseased group
HI: healthy group
NRI: net relatedness index
PCoA: principal coordinate analysis
PERMANOVA: permutational multivariate analysis of variance
SIMPER: similarity percentage analysis
βMNTD: the pairwise phylogenetic turnover between communities
βNTI: β-nearest taxon index
