# Supplementary material for: Disease outbreak accompanies the dispersive structure of shrimp gut bacterial community with a simple core microbiota
Source: AMB Express. 2018 Jul 18;8:120. doi: 10.1186/s13568-018-0644-x (PMC6051950; doi:10.1186/s13568-018-0644-x)
Supplement: Supplementary file 1 — Additional file 1: Table S1. The average body length and the weight of the shrimp under different health state across three sampling days. Figure S1. Rarefaction curves of individual shrimp samples. Rarefaction curves were assembled showing the number of OTUs, defined at the 97% sequence similarity cut-off, relative to the number of total sequences. The dashed vertical line indicates the number of sequences subsampled from each sample to calculate alpha diversity estimates. Figure S2. The alpha-diversity indices (Shannon index and number of observed species, per 26100 sequences of the shrimp gut samples under different health state across three sampling days. HI: healthy gut; DI: diseased gut. Significant differences were indicated by the asterisk (*, P < 0.05) based on one-way analysis of variance. Lines at the top, bottom, and middle of the box correspond to the 75th, 25th, and 50th percentiles (median), respectively. The asterisk in the box represents the mean value. Figure S3. Relative abundance of the dominant bacterial phyla (>3%) or classes (Proteobacteria) under different health state across three sampling days. Each bar represents the mean ± standard deviation. Significant differences were indicated by the asterisk (*, P < 0.05; **, P < 0.01) based on one-way analysis of variance. Figure S4. Principal coordinate analysis (PCoA) plots of community dissimilarities based on unweighed Unifrac distance between healthy and diseased shrimp gut across three sampling days. Sampling days exhibited with distinct colors (Blue: Day 70; Red: Day 80; Green: Day 85) and health state showed with the solid (Healthy) and hollow (Diseased). Permutational Multivariate Analysis of Variance (PERMANOVA) was used to test the significance of time, health state and their interaction in community variation at each day. HI: healthy gut; DI: diseased gut. [file 13568_2018_644_MOESM1_ESM.docx]

**AMB express**

**Supplementary material**

**Disease outbreak accompanies the dispersive structure of shrimp gut bacterial community with a simple core microbiota**

Zhiyuan Yao^1^, Kunjie Yang^1^, Lei Huang^1^, Xiaolin Huang^1,2^, Linglin Qiuqian^1^, Kai Wang^1,3^, Demin Zhang^1,3*^

^1^School of Marine Sciences, Ningbo University, Ningbo, 315211, China

^2^Zhejiang Mariculture Research Institute, Wenzhou, 325005, China

^3^Collaborative Innovation Center for Zhejiang Marine High-efficiency and Healthy Aquaculture, Ningbo, 315211, China

Zhiyuan Yao: [yaozhiyuan@nbu.edu.cn](mailto:yaozhiyuan@nbu.edu.cn)

Kunjie Yang: 317762492@qq.com

Lei Huang: huanglein@sina.com

Xiaolin Huang: 53706069@qq.com

Linglin Qiuqian: 1026483115@qq.com

Kai Wang: wangkai@nbu.edu.cn

Demin Zhang: zhangdemin@nbu.edu.cn

*For correspondence. E-mail: zhangdemin@nbu.edu.cn (Demin Zhang); Tel. 86-574-87600164; Fax 86-574-87608347

Table S1 The average body length and the weight of the shrimp under different health state across three sampling days

| Sampling day | Health status | Number | Length/cm | Weight/g |
| --- | --- | --- | --- | --- |
| D70 | HI | 14 | 5.79±0.52^a^ | 7.36±0.42^a^ |
|  | DI | 12 | 5.10±0.80^a^ | 7.10±0.61^a^ |
| D80 | HI | 9 | 7.70±1.70^a^ | 8.60±0.80^a^ |
|  | DI | 6 | 6.61±2.22^a^ | 8.38±0.82^a^ |
| D85 | HI | 8 | 8.63±2.56^a^ | 9.12±0.48^a^ |
|  | DI | 9 | 7.65±1.78^a^ | 8.58±0.71^b^ |

Different letters denote significant differences (*P* < 0.05) between health states on the same sampling day. Means ± standard deviations were compared using one-way analysis of variance (ANOVA).

Figure S1 Rarefaction curves of individual shrimp samples. Rarefaction curves were assembled showing the number of OTUs, defined at the 97% sequence similarity cut-off, relative to the number of total sequences. The dashed vertical line indicates the number of sequences subsampled from each sample to calculate alpha diversity estimates.


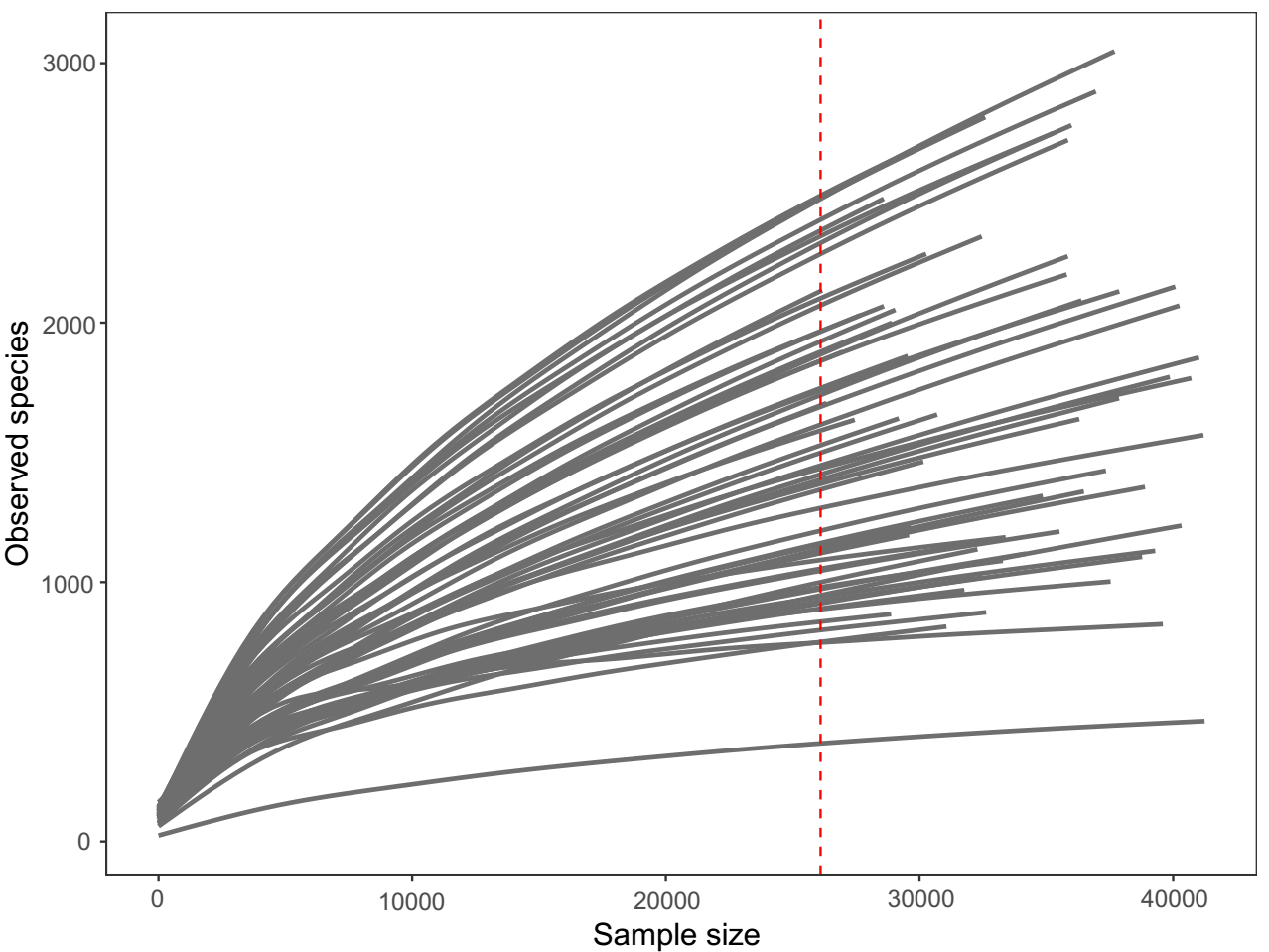





Figure S2 The alpha-diversity indices (Shannon index and number of observed species, per 26100 sequences of the shrimp gut samples under different health state across three sampling days. HI: healthy gut; DI: diseased gut. Significant differences were indicated by the asterisk (*, *P* < 0.05) based on one-way analysis of variance. Lines at the top, bottom, and middle of the box correspond to the 75th, 25th, and 50th percentiles (median), respectively. The asterisk in the box represents the mean value.


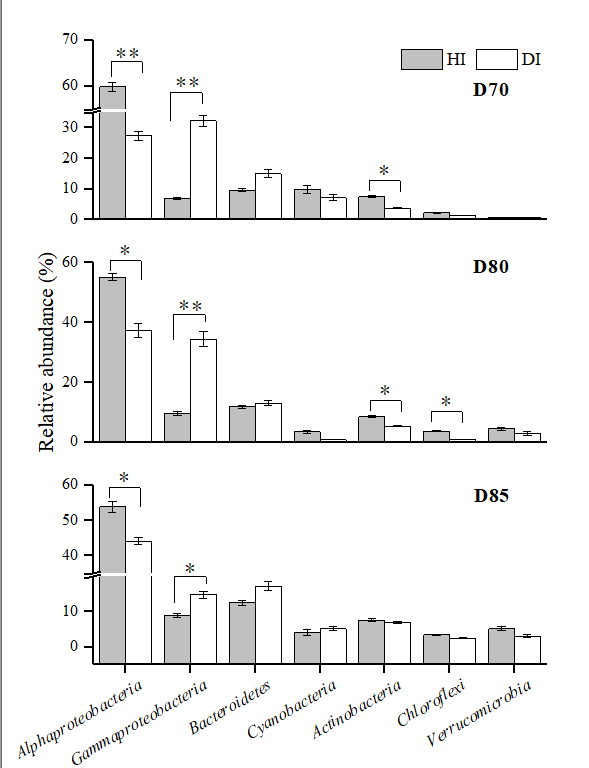


Figure S3 Relative abundance of the dominant bacterial phyla (>3%) or classes (*Proteobacteria*) under different health state across three sampling days. Each bar represents the mean ± standard deviation. Significant differences were indicated by the asterisk (*, *P* < 0.05; **, *P* < 0.01) based on one-way analysis of variance.


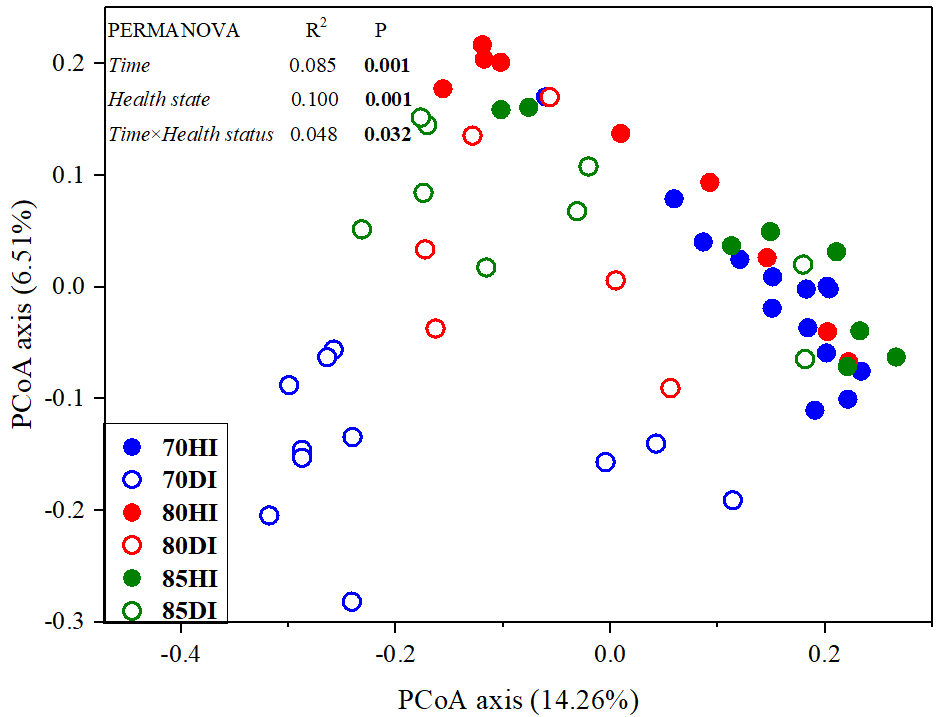


Figure S4 Principal coordinate analysis (PCoA) plots of community dissimilarities based on unweighed Unifrac distance between healthy and diseased shrimp gut across three sampling days. Sampling days exhibited with distinct colors (Blue: Day 70; Red: Day 80; Green: Day 85) and health state showed with the solid (Healthy) and hollow (Diseased). Permutational Multivariate Analysis of Variance (PERMANOVA) was used to test the significance of time, health state and their interaction in community variation at each day. HI: healthy gut; DI: diseased gut.
